# Predicting the effect of seine rope layout pattern and haul-in procedure on the effectiveness of demersal seine fishing: A Computer simulation-based approach

**DOI:** 10.1371/journal.pone.0182609

**Published:** 2017-08-03

**Authors:** Nina A. H. Madsen, Karl G. Aarsæther, Bent Herrmann

**Affiliations:** 1 SINTEF Fisheries and Aquaculture, Hirtshals, Denmark; 2 SINTEF Fisheries and Aquaculture, Trondheim, Norway; 3 University of Tromsø, Tromsø, Norway; Technological Educational Institute of Western Greece, GREECE

## Abstract

Demersal Seining is an active fishing method applying two long seine ropes and a seine net. Demersal seining relies on fish responding to the seine rope as it moves during the fishing process. The seine ropes and net are deployed in a specific pattern encircling an area on the seabed. In some variants of demersal seining the haul-in procedure includes a towing phase where the fishing vessel moves forward before starting to winch in the seine ropes. The initial seine rope encircled area, the gradual change in it during the haul-in process and the fish's reaction to the moving seine ropes play an important role in the catch performance of demersal seine fishing. The current study investigates this subject by applying computer simulation models for demersal seine fishing. The demersal seine fishing is dynamic in nature and therefore a dynamic model, *SeineSolver* is applied for simulating the physical behaviour of the seine ropes during the fishing process. Information about the seine rope behaviour is used as input to another simulation tool, *SeineFish* that predicts the catch performance of the demersal seine fishing process. *SeineFish* implements a simple model for how fish at the seabed reacts to an approaching seine rope. Here, the *SeineSolver* and *SeineFish* tools are applied to investigate catching performance for a Norwegian demersal seine fishery targeting cod (*Gadus morhua*) in the coastal zone. The effect of seine rope layout pattern and the duration of the towing phase are investigated. Among the four different layout patterns investigated, the square layout pattern was predicted to perform best; catching 69%-86% more fish than would be obtained with the rectangular layout pattern. Inclusion of a towing phase in the fishing process was found to increase the catch performance for all layout patterns. For the square layout pattern, inclusion of a towing phase of 15 or 35 minutes increased the catch performance by respectively 37% and 48% compared to fishing without a towing phase. These results highlights the importance of the selected seine rope layout pattern and the duration of the towing phase when fishermen try to maximize the catch performance of their fishery. To our knowledge this is the first time the combination of models for the physical behaviour of seine ropes and for fish behaviour in response to seine rope movements have been applied to predict catch performance for demersal seining.

## Introduction

**Fig 1:**
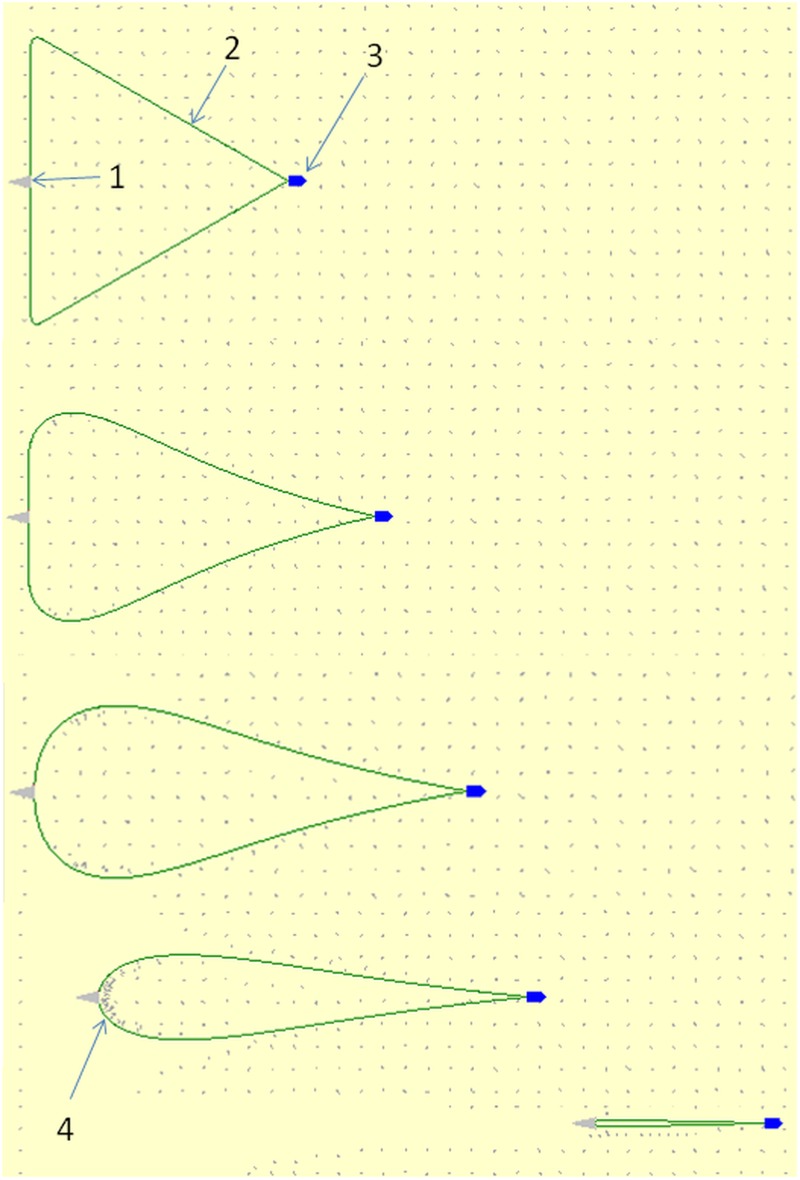
Demersal seine fishing procedure (collection and closing phase) from top to bottom. 1: seine net. 2: seine rope. 3: fishing vessel. 4: fish collected ahead of seine net. The grey dots represent aggregations of fish at the seabed. In this case, the fish is being uniformly distributed. Seine net, fishing vessel and fish aggregations are scaled up compared to the length of the seine ropes for illustration purposes.

The Danish seining or anchor seining is an active demersal fishing technique which was invented in Denmark and in the first half of the 20th century became one of the most important fishing gears used there [1]. When this fishing method was brought to other countries, it was modified to local conditions and customs. Scottish fishermen started to fish without anchoring, making it possible to move the vessel forward during hauling and thereby including a towing phase. This technique is known as Scottish seining, ‘Fly-dragging’ or ‘Fly-shooting’, and is also the method primarily applied by Norwegian fishermen targeting cod (*Gadus morhua*) and haddock (*Melanogrammus aeglefinus*) [2]. Together these variants of this fishing method can be termed as demersal seining. Today its importance as a commercial fishing method in Denmark and other parts of the world is increasing due to its low fuel consumption, high catch quality and low ecosystem impacts when compared to trawling [3–6]. For example, about 20% of the Norwegian cod quota is caught by demersal seining; the Norwegian style fly dragging [7]. Thus, knowledge about the physical behaviour of this type of fishing gear and its ability to collect fish for the seine net is important. It is relevant to investigate how effective the different variants of the demersal seining is compared to each other in particular the effect on catch performance of layout pattern deployed and by the inclusion of a towing phase and its duration. Demersal seining in Norwegian fishery targeting cod and other demersal species is practiced by deploying two long seine ropes connected to the wing tips of the seine net in one end and the winches of the vessel on the other end. The length of the seine ropes is restricted to 2000 m each when fishing inside the four nautical mile limit. The seine ropes, made of up to Ø60 mm combination rope (polyethylene with a steel core) weighing more than 2 kg/m, are placed on the seabed often in a quadrilateral pattern in order to encircle the targeted fish [8]. Once the ropes and the net have reached the seabed, the vessel starts moving forward at a speed of 1–2 knots. As a result of the vessel movement the seine ropes move towards each other and herd the fish into the centre of the encircled area; the collecting phase. At some instance, the net will start to move along the seabed being pulled by the seine ropes. When the distance between the ropes has decreased to a certain level, the rope drums are activated in order to close the wings fast and to force the last fraction of collected fish into the seine net; the closing phase. This fly dragging principle of demersal seining is shown in Fig 1.

The catching performance of demersal seine fishing depends on the area on the seabed swept and encircled by the seine ropes during the fishing process. The efficiency of the seine ropes to herd the fish into and subsequently maintain them in the path of the much smaller seine net until they are overtaken by it in the later stages of the fishing process is of similar importance for the catch performance in demersal seining. Knowledge about how the size and shape of the area encircled by the seine ropes gradually change during the fishing process and how it leads to increased density of fish is therefore important for an efficient fishery. Thus, understanding and quantifying the physical behaviour of the seine ropes and how this behaviour gradually leads to increased density of fish in the encircled area are important aspects of the demersal seine fishing process. This subject is investigated by applying simulation models for demersal seine fishing that predicts the amount of fish being collected between the seine ropes during the fishing process. The simulation method consists of combining a model for the physical behaviour of seine ropes with a simple model for fish reaction to an approaching seine rope. Results are provided for a Norwegian demersal seine fishery targeting cod in the coastal zone.

## Material and methods

This study evaluates the catch performance for 12 demersal seine fishing scenarios differing in seine rope layout pattern and haul-in procedure. For each fishing scenario, the following two steps are conducted:

1. Predicting of the kinematic behaviour of the seine ropes during the collection phase (Fig 1) of a demersal seine haul using a simulation tool *SeineSolver*.
2. Predicting of the catch performance of a demersal seine haul by using a tool *SeineFish* that simulates fish reaction to the moving seine ropes along the seabed. *SeineFish* uses the output from *SeineSolver* as input.

The material and method section is therefore structured into subsections describing: i) the method for simulating of seine rope behaviour (*SeineSolver*); ii) the model applied in *SeineFish* for simulating fish's reaction to an approaching seine rope; iii) simulating the collection phase of demersal seine fishing (*SeineFish*); and iv) the fishing scenarios investigated.

### Method for simulation of seine rope behaviour

The dynamics of the demersal seine fishing gear is dominated by the behaviour of the seine ropes. Hence, we needed a tool for the investigations that can predict the physical behaviour of the seine ropes during a demersal fishing process. We applied an existing tool hereafter named *SeineSolver*. *SeineSolver* has an interface that enables the user to specify the gear deployed including the characteristic of the seine ropes and the fishing operation in terms of layout pattern for the seine ropes, towing speed, towing time before starting winching and winching speed. *SeineSolver* uses the FhSim simulation framework [9]. The seine ropes were modelled by cables consisting of a collection of six degree of freedom elements. The cables were connected to the weight at one end, representing the seine net, and to a winch at the other. Since the demersal seine fishing is dynamic of nature a time-domain formulation of the cable dynamics is applied. The *SeineSolver* model implements the method found in [10], which includes a numerical model where the cable dynamics are described as a collection of hinged rigid bodies. *SeineSolver* uses a seabed contact model from FhSim [9] which calculates the reaction force resulting from an overlap between a cylinder element and the seabed surface. The normal force leads to a transversal friction force modelled by a friction coefficient. Time integration is performed with a simple forward Euler scheme [11] using a time-step of 0.001 sec. The model behind *SeineSolver* is thoroughly described in [12]. The ability of the *SeineSolver* model and tool to predict the kinematic behaviour of seine ropes during haul-in procedures have been tested extensively against flume tank experiments for different seine rope layout patterns and haul-in procedures. These tests have all shown that *SeineSolver* is able to accurately predict the kinematics of the seine ropes for various layout patterns and haul-in procedures [13].

### Model for fish reaction to an approaching seine rope at the seabed

**Fig 2:**
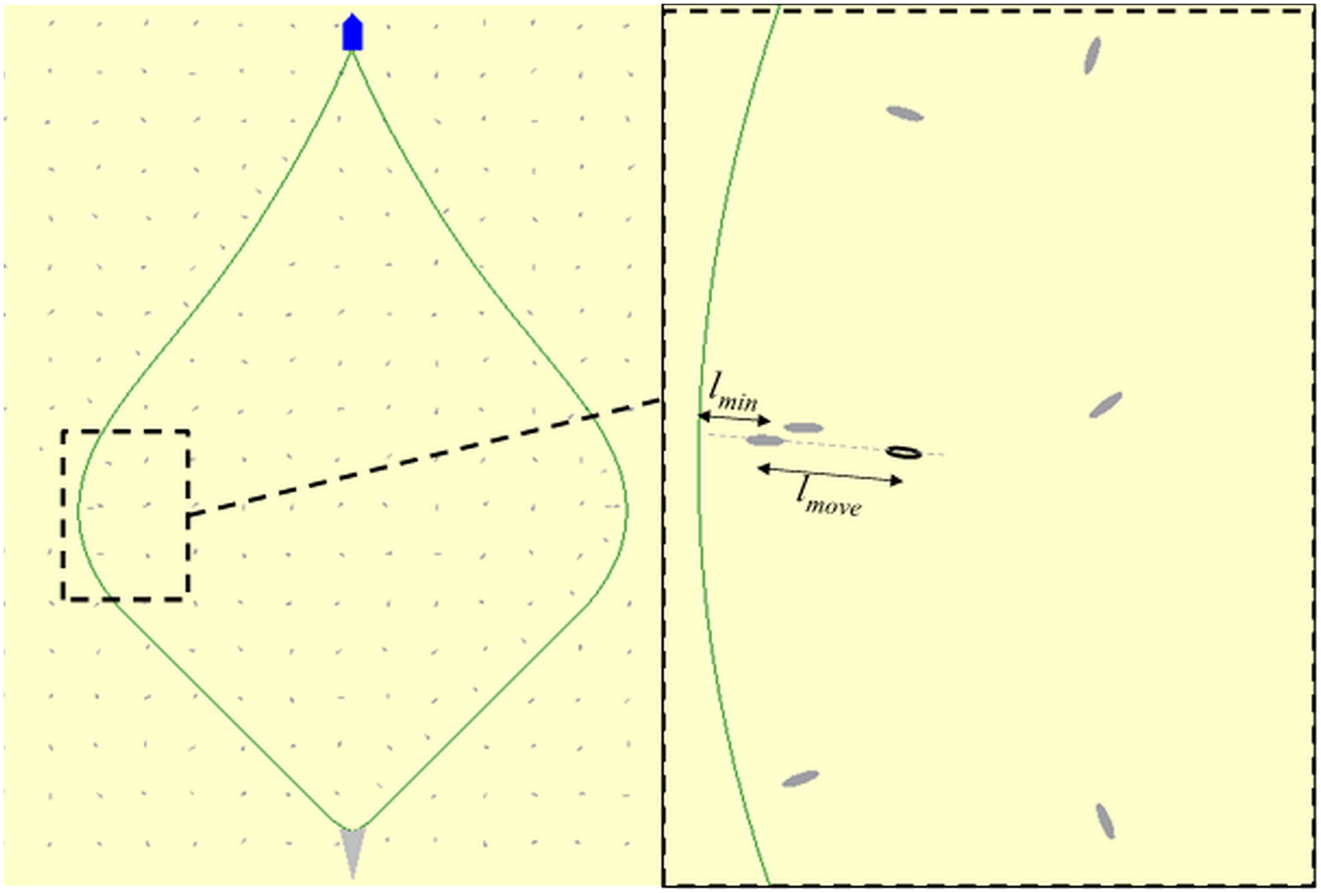
Fish (grey ellipses) reaction to an approaching seine rope (green curve). The zoomed picture on the right side illustrates that when the seine rope gets closer to the fish than the distance *l*_*min*_ it reacts by swimming the distance *l*_*move*_ further away from the seine rope in a direction perpendicular to the seine rope. The seine net, the fishing vessel and fish aggregations are scaled up compared to the length of the seine ropes for illustration purposes.

To be able to predict the effect the seine ropes have on the catch performance of demersal seine fishing by simulation we need a model for how fish near to the seabed reacts to an approaching seine rope. Little information are available for demersal seining but far more observations exists for bottom trawling. The ability of trawls sweeps on the seabed to herd cod into the centre of the trawl are demonstrated in [14]. Cod reacts with an avoidance response when the sweep wire approaches it. This can be interpreted, as the cod would keep at least some distance away from an approaching threat, in this case the sweep wire. In line with [15] it can be expected that the cod on average will react by swimming in a direction perpendicular to the approaching wire. We assume that cod reacts in a similar way to an approaching seine rope during demersal seining. Hence, we assume that if the seine rope gets closer than a distance *l*_*min*_ to the cod it will swim a distance *l*_*move*_ from its current position away from the seine rope perpendicular to the approaching rope. Fig 2 illustrates this fish behaviour to an approaching seine rope. We assume no reaction from the cod if it has as a distance to the rope that is greater than *l*_*min*_. In addition, we assume that the cod only react to the part of the seine rope on the seabed.

To account for that cod might not always react with the avoidance response along the seabed every time the seine rope gets closer than *l*_*min*_ to it, we assume that there is a small probability *p*_*raise*_ for the cod to react by raising the distance *l*_*move*_ up from the seabed for a short period, meanwhile the seine rope passes beneath it. Hence, the probability for the fish to be herded along the seabed is *p*_*herd*_ = *1.0* –*p*_*raise*_, for an incidence where the seine rope on the seabed get closer than *l*_*min*_ to it. Therefore, the cod can react in two ways to the approaching seine rope, either by a herding response or by an avoidance response. The herding response is modelled by the cod moving a distance *l*_*move*_, along the seabed further away from and perpendicular to the seine ropes. The avoidance response is modelled by letting the seine rope pass beneath the fish. For each incident, where the distance from the fish to a seine rope becomes smaller than *l*_*min*_ one of these responses is triggered. The probability of triggering each of these responses is modelled by a binomial process with probabilities *p*_*herd*_ and *p*_*raise*_. Underwater recordings conducted in Norwegian bottom trawl fishery shows cod often try to maintain a distance of 1–2 m ahead of the ground rope. Therefore, we assume *l*_*min*_ to be 1.5 m. In addition, we assume *l*_*move*_ to be twice *l*_*min*_. For simplicity and explorative purposes, we assume that cod reacts with a herding response each time the seine rope gets too close to it. This means we will fix *p*_*herd*_ at 1.0.

### Simulating the collection phase of demersal seine fishing

The model for fish reaction to an approaching seine rope was implemented in the software tool *SeineFish*. *SeineFish* has a graphical user interface and runs on a personal computer with Microsoft Windows operating system (version 7 or later). *SeineFish* simulates the collecting phase in a demersal seining haul. To do so *SeineFish* uses externally generated information on the physical behaviour of the seine ropes. This information is obtained with *SeineSolver*. The *SeineSolver* output file contains information on the kinematics of the seine ropes and seine net position for a simulated demersal seine fishing process. Specifically for discrete steps in time, the *SeineSolver* output file contains positions for points along the seine ropes during the simulated fishing process. Based on this information *SeineFish* models the geometry of the front part of the demersal seine gear continuously in time and space by using a nested linear interpolation technique. In the outer interpolation loop, poistions for the seine rope points for two adjacent time steps *t*_*1*_ and *t*_*2*_ are read into two polygons. Then by linear interpolation, a polygon at arbitrary time *t* between *t*_*1*_ and *t*_*2*_ is predicted. Next, in the inner interpolation loop, the geometry of the front part of the gear is predicted for arbitrary positions along the gear by linear interpolation using the polygon points at time *t*. Prior to starting the simulated fishing in *SeineFish* the user defines a virtual fish population and its spatial distribution on the virtual fishing ground. The distribution and the density of fish on a fishing ground varies between individual demersal seine hauls, hereafter called "fishing operations". In this study we are interested in investigating the average catch performance obtained over multiple fishing operations by deploying one specific seine rope layout pattern and one specific haul-in procedure, "a fishing case", compared with the catch performance for another fishing case. One obvious way of obtaining comparable results for the different fishing cases would be for each case to simulate the same large number of different fishing operations with different spatial fish distributions and densities and then estimate the average catch for each fishing case to enable an average comparison between fishing cases. However, when averaging over multiple hauls the probability to initially find a fish at a specific position on the fishing ground will be uniformly distributed. Therefore, it is sufficient for the purpose of comparing the average catch performance to run one simulation for each fishing case assuming a uniform fish distribution on the fishing ground even through this may not be a realistic scenario for the individual fishing operations. Besides the distribution pattern, the user also input the value *fish*_*dens*_ (number of fish per m^2^ fishing ground) which defines the average density of fish on the fishing ground. For all the simulations in this study we set *fish*_*dens*_ at 0.01 m^2^ corresponding to on average 100 fish for each 10000 m^2^. This value was considered realistic based on total catches of cod obtained during typical demersal seine fishing in Norwegian coastal zone. However, based on similar consideration as above, the actual value selected for *fish*_*dens*_ should not affect the relative catch performance between fishing cases as long as the same value have been applied for all cases. During the simulated fishing process, the distribution pattern of the fish will gradually change due to their interaction with the fishing gear. This interaction is simulated by the fish reaction model and governed by the values chosen by the user for the parameters *l*_*min*_, *l*_*move*_ and *p*_*herd*_.

The simulation of the fishing process in *SeineFish* can be characterized as a time-step integration technique (time step = 0.2 sec) where the position and shape of the seine gear on the fishing ground is updated and the interaction with the individual fish is simulated according to the procedure described above. During the simulation, the value for key indicators are calculated and logged. The indicators are: the area encircled by the part of the seine ropes on the seabed (*A*_*encirled*_ [m^2^]); entry width of the gear (*w*_*entry*_ [m]) being the horizontal distance across the fishing ground between the two points closest to the fishing vessel on respectively the right and left seine rope that has contact with the seabed; and finally the number of fish (*fish*_*encirled*_) in the encircled area on the seabed. The simulated fishing process is continuously visualized in *SeineFish* by illustrating the fishing gears shape and position as well as the position and movement of the fish caused by their reaction to the fishing gear. Fig 1 and several of the following figures in this paper have been created based on screen dumps during simulations using *SeineFish*.

### Fishing scenarios

**Fig 3:**
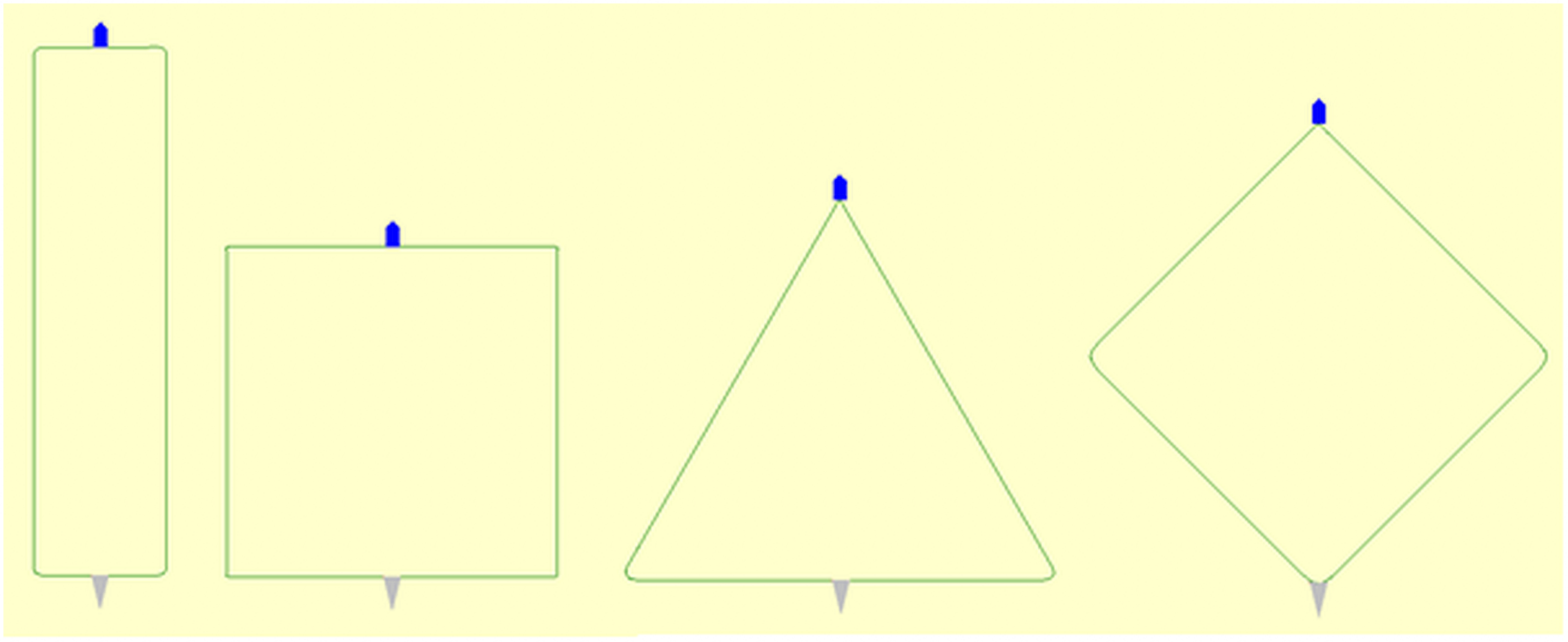
The four different initial layout patterns simulated. From left: rectangular, square, triangular and diamond. The seine net (grey triangle) and the fishing vessel (blue pentagon) are scaled up compared to the extent of the seine ropes for illustration purposes (screen dumps from simulations using *SeineFish*).

To investigate the potential effect of initial seine rope layout pattern on the catch performance for demersal seining targeting cod in coastal zone in Norwegian fishery we investigated four different initial layout patterns: rectangle, square, triangle and diamond (Fig 3).

**Fig 4:**
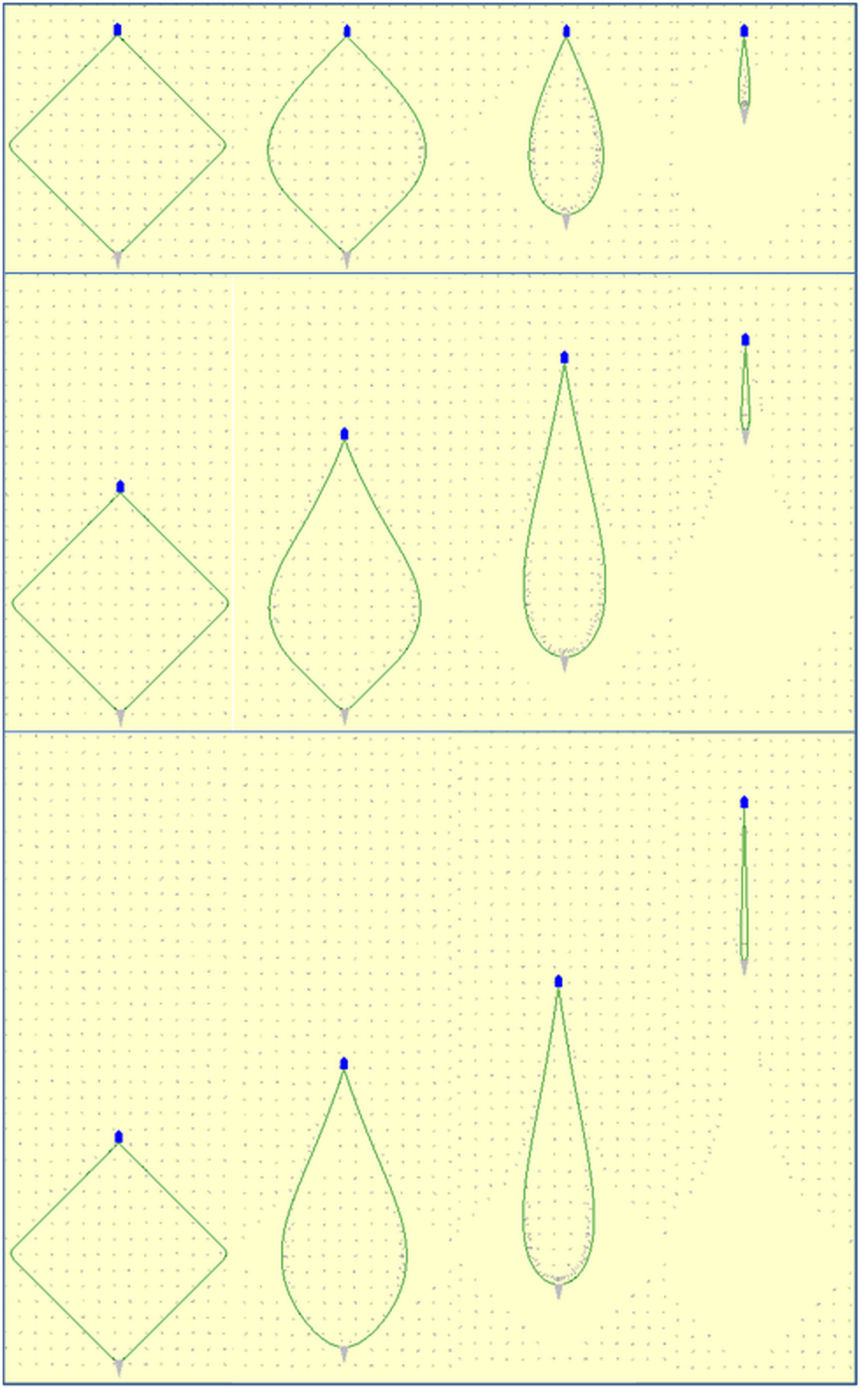
Illustration of the fishing process (from left to right) for each of the three towing phase cases investigated. From top: no towing, 15 minutes towing and 35 minutes towing. Here illustrated for the diamond shaped initial layout pattern. The seine net (grey triangle), the fishing vessel (blue pentagon) and thefish aggregations (grey dots) are scaled up compared to the length of the seine ropes (green curves) for illustration purposes (screen dumps from simulations using *SeineFish*).

Two seine ropes each of approximately 2000 m were laid out initially for all layout patterns. As such complying with the legislation for the Norwegian coastal fishery and enabling a fair comparison between cases. The seine rope diameter was 36 mm as typically used in this fishery. Each layout pattern was deployed with three different haul-in procedures to enable investigating the effect on catch performance by haul-in procedure. The three haul-in procedures differs by the time the vessel was towing before starting to winch the seine ropes, respectively 0, 15 and 35 minutes. The first case without towing represents the original Danish seine or anchor seine fishing while the two other represents Scottish seining or fly-dragging. The towing speed was two knots and the winching speed 0.9 m/s, which are settings also applied commercially in this fishery. Fig 4 illustrates the three towing phase cases investigated.

For each of the 12 fishing cases (four different initial layout cases times three different haul-in procedures) we first used *SeineSolver* to estimate the physical behaviour of the front part of the fishing gear (seine ropes) during the simulated fishing process. The predicted gear behaviours were subsequently used as input in *SeineFish* to simulate the collection phase for the demersal seine for each of the 12 fishing cases. Since identical fish populations were used for the different fishing cases, we could use the values for the encircled number of fish as a relative measure for the effectiveness of the fishing process for the different cases. In addition to monitoring the number of fish encircled during the simulated process, we also monitored the size of the encircled area and the entry width between the seine ropes. The entry width is important for the effectiveness of a towing phase because it is only through this opening that the number of encircled fish can increase.

## Results

### Simulating fishing scenarios

**Fig 5:**
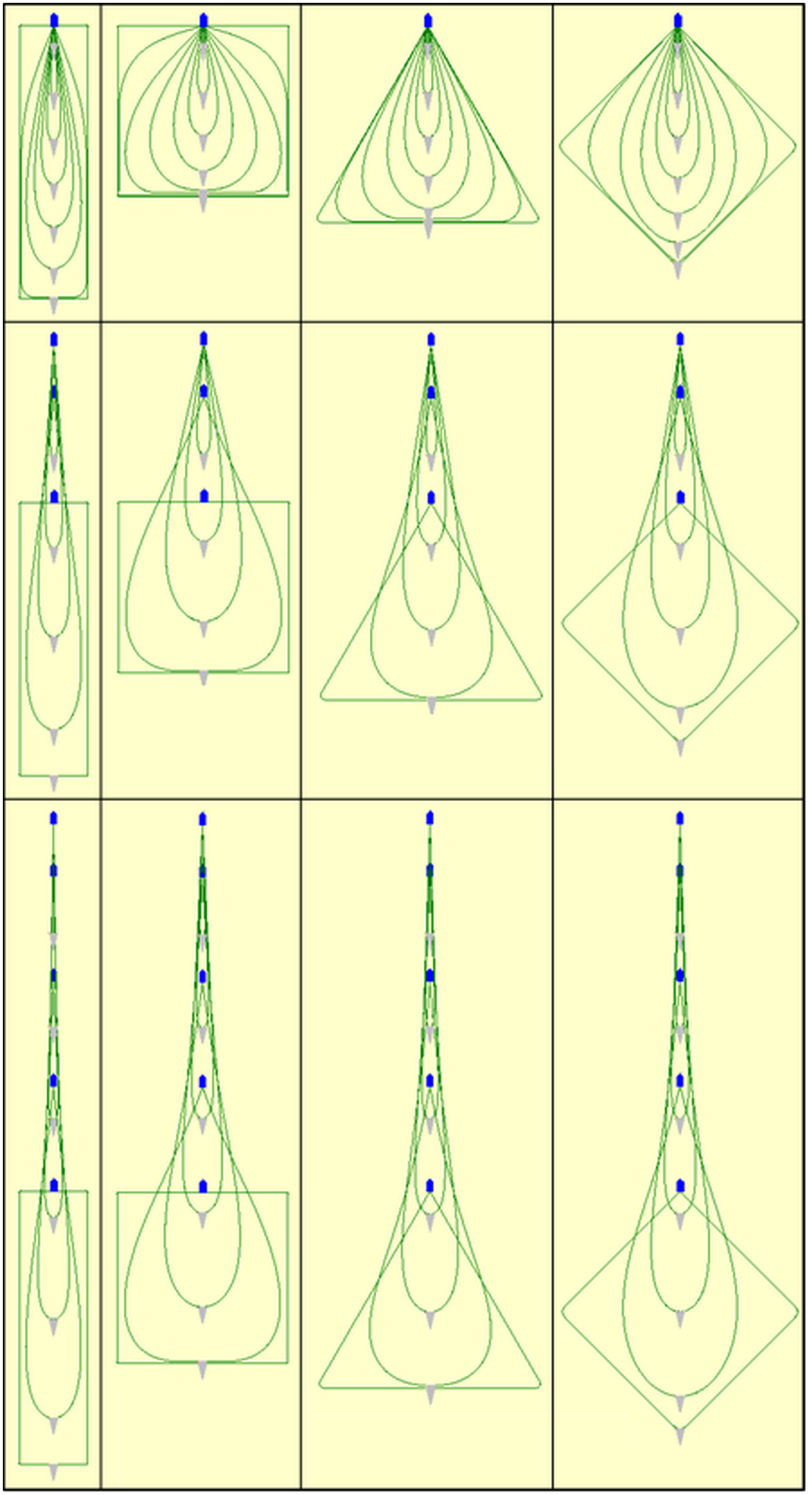
Illustration of the physical behaviour of the fishing gear during different steps of the fishing process for each of the 12 fishing cases investigated. From top to bottom: no towing, 15 minutes towing, 35 minutes towing. From left to right: rectangular, square, triangular, diamond initial layout pattern. The seine net (grey triangle) and the fishing vessel (blue pentagon) are scaled up compared to the length of the seine ropes (green curves) for illustration purposes (screen dumps from simulations using *SeineFish*).

Fig 5 illustrates the physical behaviour of the fishing gear during steps in the fishing process for each of the 12 simulated fishing processes.

### Number of fish encircled

**Fig 6:**
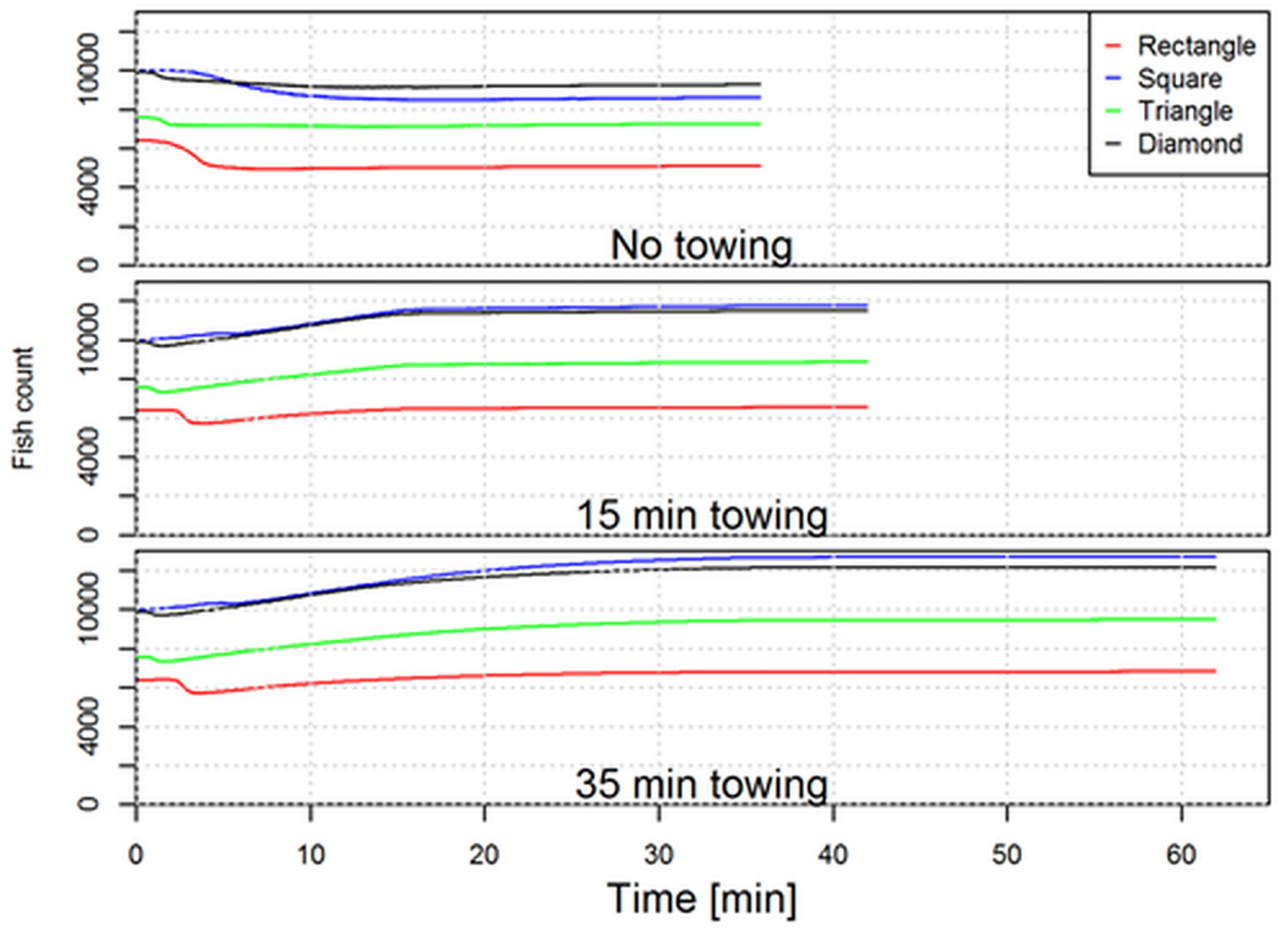
Development in the number of fish encircled by the seine ropes on the seabed (fish count) during the fishing process for deployment of the four initial layout patterns investigated and for each towing phase scenarios.

The *SeineSolver* and *SeineFish* tools were applied to predict the number of fish encircled during the fishing process for each fishing case. From Fig 6 it is evident that for the same seine rope length being deployed on the fishing ground, in this case 2 x 2000 m, the number of fish being encircled by the seine ropes depends strongly on the initial layout pattern. Specifically we see that the square and diamond layout patterns are predicted to encircle a much higher number of fish than for the triangular and in particular the rectangular pattern.

Fig 6 illustrates the increase in the number of encircled fish as the towing phase progresses and demonstrates the benefit of a long towing phase (35 minutes). Table 1 quantifies for each of the 12 fishing cases the number of fish being encircled initially, when winching begins and at the end of the fishing process.

**Table 1:** Number of fish encircled during the fishing process. Numbers in parenthesis are percentage increase compared to value after initial layout.

Layout pattern	Towing time [minutes]	Number of encircled fish		
		Initial	At start of winching	At end of process
Rectangle	0	6399	6399 (0%)	5098 (-20%)
Rectangle	15	6399	6458 (1%)	6564 (3%)
Rectangle	35	6399	6813 (6%)	6837 (7%)
Square	0	9999	9999 (0%)	8605 (-14%)
Square	15	10000	11505 (15%)	11783 (18%)
Square	35	10000	12684 (27%)	12739 (27%)
Triangle	0	7581	7581 (0%)	7270 (-4%)
Triangle	15	7582	8681 (14%)	8881 (17%)
Triangle	35	7582	9456 (25%)	9498 (25%)
Diamond	0	9897	9897 (0%)	9270 (-6%)
Diamond	15	9897	11319 (14%)	11526 (16%)
Diamond	35	9897	12150 (23%)	12192 (23%)

The rectangular and triangular layout patterns are predicted to initially encircle respectively only 64% and 76% of the number of fish being encircled with the square and diamond layout patterns (Table 1). At the end of the fishing process this difference is increased further and depends also on which of the three simulated haul-in procedures that has been applied. Based on the values in Table 1 it can for example be calculated that for respectively 0, 15 and 35 minutes towing before winching that the square layout end up encircling respectively 69%, 80% and 86% more fish than is predicted to be obtained with the rectangular layout pattern. The effect of respectively 15 or 35 minutes towing compared to a fishing process without a towing phase can also be calculated from Table 1. For the rectangular layout pattern a 15 minutes towing phase results in 29% increase in catch while the increase is 34% with 35 minutes towing. For the square layout pattern 15 or 35 minutes towing are predicted to increase catch by respectively 37% and 48%. With the triangle layout pattern the predicted catch increase are respectively 22% and 31%, while it for the diamond layout pattern are 24% and 32%. In general, it is found that without towing the number of encircled fish will decrease from the initial value with a percentage that depends on the initial layout pattern.

### Area encircled on the seabed by the seine ropes

**Fig 7:**
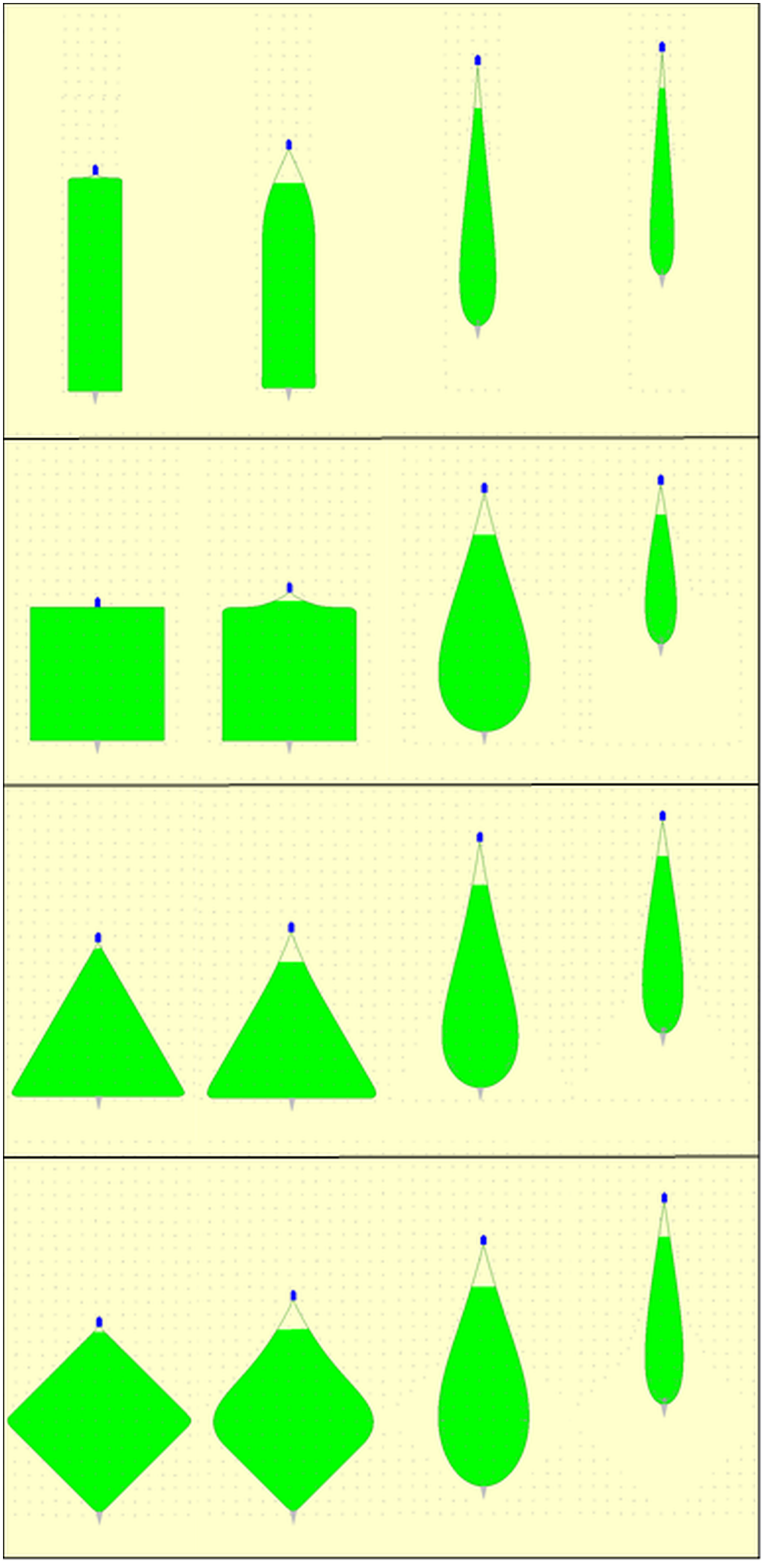
Illustration of the development in the area encircled (green area) by the seine ropes on the seabed during the fishing process (from left to right) for the four layout patterns (from top to bottom). Here illustrated for the 15 minutes towing phase. The seine net (grey triangle), the fishing vessel (blue pentagon) and the fish aggregations (grey dots) are scaled up compared to the length of the seine ropes (green curves) for illustration purposes (screen dumps from simulations using *SeineFish*).

To help understand the difference in performance of the layout patterns regarding their ability to encircle fish during the fishing process it can be useful to look at the development of the geometrical properties for the gear during the fishing process. Fig 7 illustrates for a towing phase of 15 minutes the development in area encircled by seine ropes on the seabed (green filled areas on Fig 7).

**Fig 8:**
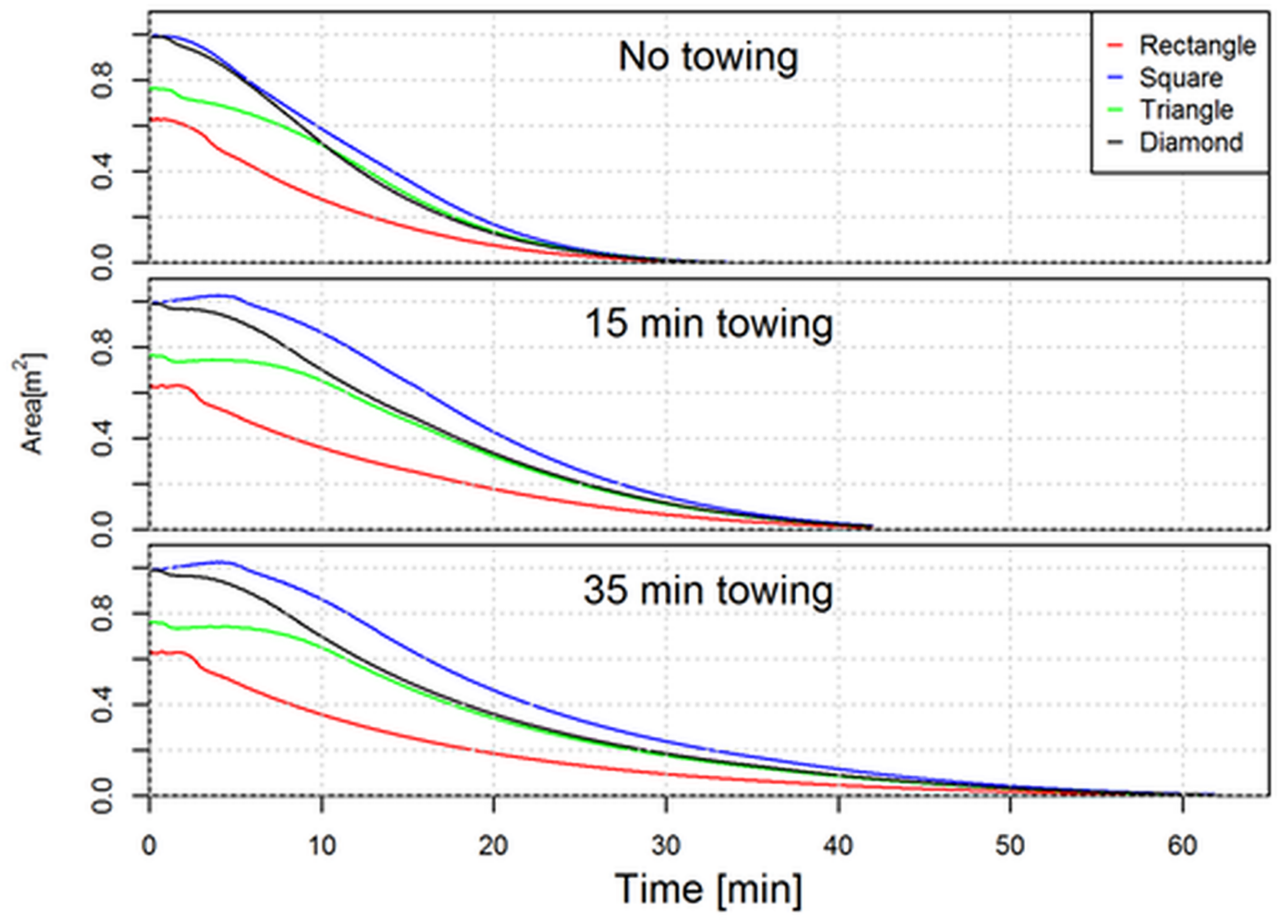
Development in the area encircled by the seine ropes on the seabed during the fishing process for the four initial layout patterns investigated and for each towing phase scenarios.

The difference in the development in the encircled area for the different initial layout patterns during the fishing process is clear (Fig 7). The development in entry width (where the seine ropes are lifted from the seabed) into the encircled area is also seen in Fig 7 and the narrowness of it is clear. This illustrates the challenge of benefitting from a long towing phase, but also why a short towing phase benefits the catch compared to not towing at all. The seine ropes are pulled at by the vessel causing parts of the seine ropes to lift of the seabed leading to a decrease in the encircled area and thereby of the collected fish. During the first part of a towing phase, this amount of fish is regained through the entry width and the area covered through this. This phenomenon is also clear from Fig 6 which shows the decrease in the number of fish encircled when the vessel starts pulling at the seine rope. Without a towing phase (Fig 6 top) this loss is never regained during the remaining fishing process. Contrary with a towing phase of 15 or 35 minutes this loss is regained (Fig 6 middle and bottom). Fig 8 quantifies the development in the encircled area during the fishing process for each of the 12 fishing cases investigated.

**Fig 9:**
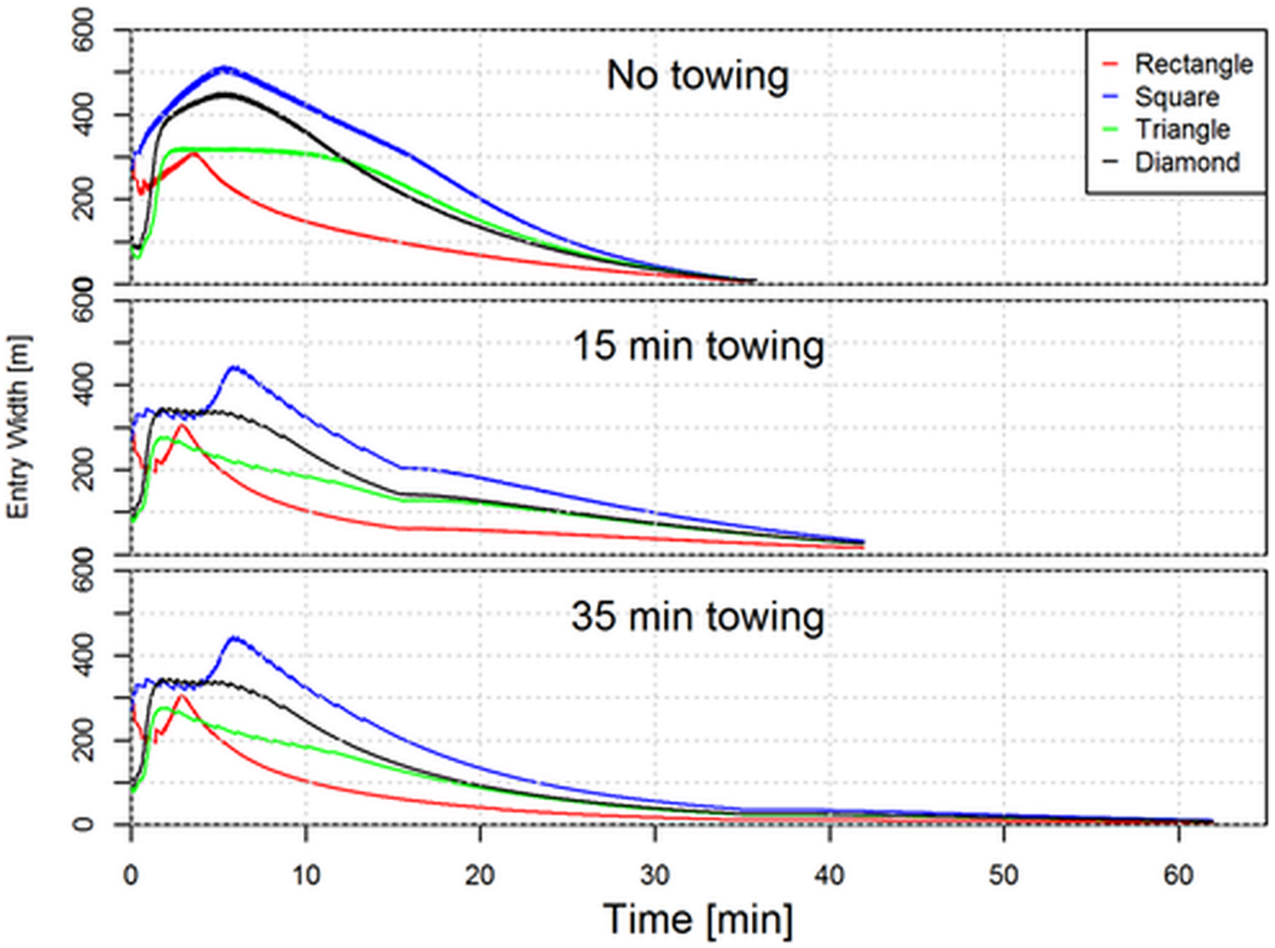
Development in the entry width into the area encircled by the seine ropes on the seabed during the fishing process for the four initial layout patterns investigated and for each towing phase scenarios.

From Fig 8 it is clear the initially encircled area depends strongly on the layout pattern applied and as expected we can fully explain the differences in initial number of fish being encircled between the different layout patterns (Table 1). It is seen for a fishing process without a towing phase (Fig 8 top) that the encircled area diminishes earlier than if a towing phase of some duration was included in the fishing process (Fig 8 middle and bottom). However to understand the increase in number of fish encircled during the fishing process we need to look on another geometrical indicator for the gear. We have to look on the entry width to the encircled area since it is through this that additional fish enters the encircled area when the seine ropes are dragged forward to cover additional area on the seabed. Fig 9 quantifies the entry width during the fishing process.

From Fig 9 is it evident that the predicted entry width for much of the fishing phase is far smaller for the rectangular layout when compared to the other layouts and in particular with the square. This provides a potential explanation for why the predicted increase in number of fish encircled increase far less for this layout compared to each of the other layouts (see Table 1). It is interesting to see that the initial entry width is big for the rectangular layout but quickly decreases while the opposite happens for the diamond layout.

## Discussion

In this study, we have investigated how the catch performance for a demersal seine fishing operation may be affected by the initial seine rope layout pattern and by the haul-in procedure. Specifically, we have investigated the effect of including a towing phase of some duration since this is one of the major differences between the variants of the demersal seine fishing method. Our study was based on applying sequentially two different simulation models. First, *SeineSolver* estimates the physical behaviour of the seine ropes and second *SeineFish* uses this output to simulate fishing when the gear is deployed on a virtual fishing ground with a prescribed fish population distributed on it. *SeineFish* implements a simple model for how cod is assumed to react to an approaching seine rope dragged over the seabed during a demersal seine fishing operation. This model may be too simplistic, but we expect that it anyway will estimate fairly realistic how different layout patterns and haul-in procedures may affect the catching effectiveness of a demersal seine at least relative to each other. Further, this behavioural model can easily be made more complex by e.g. considering endurance of the fish after they have been forced to swim over some distance. An easy way to implement this would be to make *p*_*herd*_ a decreasing function of the total distance the fish has been forced to swim. Further *p*_*herd*_ can be made dependent on the size of the fish.

One obvious advantage of using simulation for our study is that we control what is on the fishing ground. Specifically, this means that we were able to test the different fishing cases on identical fishing conditions with respect to number of fish and spatial distribution on the fishing ground, which is essential for being able to conduct a fair comparison between the different fishing cases tested. It further provides a cheap and fast method for exploring how different aspects can affect the effectiveness of demersal seine fishing. In this study, we found that the effectiveness of demersal seining in the Norwegian coastal zone targeting cod will depend on the seine rope layout pattern applied. Specifically, we predict that the square layout pattern catch 69–86% more cod than the rectangular layout pattern often applied in the commercial fishery [16]. This highlights the importance of considering initial layout pattern when planning demersal seine fishing. Our results also demonstrated that the length of the towing phase can significantly affect the total catch but that the extent depends on the layout pattern applied. These results should be of interest for fishermen and they might help them improve catch efficiencies in demersal seining especially when considering as referenced above that it is not uncommon to apply a rectangular seine rope layout pattern in the commercial fishery. Our method allows predicting the span of results for catch performance with a specific demersal seine gear dependent on how the fishermen deploy the gear regarding seine rope layout pattern and haul-in procedure. Therefore, our method can provide guidelines to fisheries managers regarding how demersal seine catch performance depend on technical regulations like maximum seine rope length and gear deployment procedures.

In this study, we have focused on investigating the effect on the relative catch performance between different fishing cases averaged over multiple fishing operations. For this purpose, it was possible to obtain comparable results by running one simulation for each fishing case assuming a uniform spatial distribution of cod on the fishing ground and assuming one specific density of fish. However, based on the method we have described in this study it also will be possible to explore how differences in fish's spatial distribution and density affects catch performance for different fishing cases. However, such an investigation is outside the scope of the current study.

In this study, we have investigated the catch performance for different idealized seine rope layout patterns that all are symmetrical. However, some demersal seine fisheries can also apply more complicated layout patterns. One such case is found in the Danish anchor seine fishery often targeting the flatfish species plaice (*Pleuronectes platessa)* and witch flounder (*Glyptocephalus cynoglossus*). Here fishermen often use an asymmetric seine rope layout pattern and a towing phase that only drag the second seine rope to return the vessel to the buoy at the anchor [17]. In such cases, it is more complicated to predict the effect on the catch performance for the fishing operation dependent on how the fishing process is planned and conducted. However, the method we have described and applied in this study also have the potential to investigate such fisheries.

Simulation models have previously proven to be useful for predicting fish capture with active fishing by combining models for the physical behaviour of the fishing gear with models for fish behaviour to the gear [18, 19, 20]. To our knowledge those models have focused on trawls and mainly size selectivity in codends. One such model for codend is the selectivity simulator PRESEMO [21] which have used input about the physical behaviour of the gear from respectively the model of Priour [22] or the model of O'Neill [23]. To our knowledge, this is the first time that such combination of physical and behavioural models have been applied to investigate aspects of effectiveness of demersal seining.
